# Factors associated with public knowledge of and attitudes to dementia: A cross-sectional study

**DOI:** 10.1371/journal.pone.0210543

**Published:** 2019-02-28

**Authors:** Michael Rosato, Gerard Leavey, Janine Cooper, Paul De Cock, Paula Devine

**Affiliations:** 1 Bamford Centre for Mental Health and Wellbeing, School of Psychology, Ulster University, Coleraine, Northern Ireland; 2 School of Social Sciences, Education and Social Work, Queen’s University Belfast, Belfast, Northern Ireland; Università degli Studi di Perugia, ITALY

## Abstract

**Introduction:**

Dementia is a major public health concern but one that continues to be stigmatised. We examine lay knowledge of dementia and attitudes to people with dementia as potential precursors of public anxiety, focusing on the social characteristics associated with (a) the formation of these attitudes, and (b) the perception of the need for restriction and control for people with dementia.

**Methods:**

Analysis of the 2014 Northern Ireland Life and Times survey, which included questions on knowledge of, attitudes to and personal experience with dementia. We used (a) latent class analysis and (b) logistic regression to examine factors associated with respondent attitudes towards dementia.

**Results:**

Respondents (n = 1211) had relatively good general knowledge of dementia, but limited knowledge of specific risk factors. Negative perceptions of dementia were mitigated somewhat by personal contact. A high proportion of respondents felt that high levels of control were appropriate for people diagnosed with dementia, even at early stages of the disease.

**Conclusion:**

Personal antipathy to dementia was highly prevalent despite ongoing public campaigns to increase public awareness of developments in its prevention, treatment and consequent care pathways and hampering efforts to widen social inclusion. Fresh thinking and more resources may be needed to challenge persisting common misapprehension of the condition and the formation of entrenched stigma.

## Introduction

Although approximately 30% of people who reach the age of eighty are likely to have some form of dementia [1] evidence suggests that public knowledge of the disease is limited [2–4]. While initiatives such as dementia friendly health-care settings and communities [5,6] seek greater social inclusion for people with dementia there is scant evidence of their success. The World Health Organization has called for national public health agendas to highlight dementia as a priority, especially in relation to prevention, early detection and intervention [7–9], and including calls for greater public awareness of dementia in general, and increasing lay knowledge of modifiable risk factors [10,9]: improvement in both are key to reducing dementia-related stigma.

Current research in dementia-related stigma reveals social isolation [11] and discrimination, even within medical care settings [12]. It may partially explain deficits in rates of early dementia diagnosis in the United Kingdom [13] acting as a barrier to help-seeking [14], emotional and psychological distress and social exclusion. Werner *et al* [15] suggest that stigma felt by family caregivers increased their negative experience of caregiving.

Stigma is the assigning of a *socially discrediting* stereotype by the wider society which provokes an individual to feel rejected in some way—a *spoiled identity* [16]. The concept has a long history in social research and has been applied to analysis of, for example, ethnicity, sexuality and health-related contexts such as human immunodeficiency virus (HIV/AIDS) and disability [17]. In recent years there has been increasing public awareness about dementia [18] including a trend for more in-depth sympathetic representations [19]. However, anxiety about dementia-related agitative behaviour may result in persisting high levels of social exclusion [20, 21, 22]. Additionally, an emphasis in institutional care settings on containment and control, rather than person-centred approaches, can be seen as reasonable and therefore accep and more likely. Surveillance or *assistive* technology is promoted as a means of monitoring people who exhibit agitative behaviour, including *wandering–*a distressing, poorly understood problem often the reason for nursing home admission [23,24]: a solution maybe, but one with significant ethical and human rights implications [25]. This study examines both current public knowledge of dementia and attitudes to people with dementia using the Northern Ireland Life and Times Survey [26]. In particular, we examine the socio-demographic characteristics associated with (a) lay knowledge of dementia, (b) lay assumptions about dementia, and (c) attitudes to control and coercion thought appropriate for people with dementia.

## Materials and methods

### Study setting, design and participants

The Northern Ireland Life and Times (NILT) survey is a cross-sectional attitudinal survey, undertaken annually in Northern Ireland (NI) since 1998, involving a random sample of 1211 adults aged eighteen years or more. Full details of the survey design and methodology are outlined in the published Technical Notes [26]. Briefly however, a two-stage sampling process was used, as a comprehensive sampling frame of individuals was not available to the researchers: a systematic random sample of 2,449 addresses was selected from the Northern Ireland Postcode Address File (with business addresses excluded); and, using the *next birthday rule*, the interviewee was randomly selected from those aged eighteen or over living at the address. The main fieldwork was carried out from 22/09/2014 to 26/12/2014. More general notes on the survey are available elsewhere [27,2]. Two questionnaires were devised for use in the 2014 survey—both using an Ipad for data entry: a main face-to-face interview; and a shorter self-completion questionnaire for more sensitive issues (although the interviewer could record responses if requested). Both are accessible from the main NILT website (www.ark.ac.uk/nilt), and this analysis is based on the former. Because the question modules change from year-to-year ethical approval is obtained annually: the 2014 survey received ethical approval from the Ethics Committee in the School of Sociology, Social Policy and Social Work, Queen’s University Belfast (http://www.ark.ac.uk/nilt/2014/).

Data comprised a number of questions assigning socio-demographic attributes–including: age (grouped for this analysis as 18–34, 35–54, 55–74 and 75 years or older); gender; highest educational qualification (*degree-level*, *intermediate* level, and *no stated qualifications*); occupational social class (*professional*, *intermediate*, *routine social classes*, and *other*–i.e. persons classified to a social class by means other than occupation); economic activity (summarised as *active*, *inactive*); personal and household income (separately, each grouped into four bands from highest to lowest income levels); marital status (*currently married/cohabiting*, *never married*, with those *widowed*, *separated or divorced* combined as a single category); housing tenure (*owner occupier*, *private renter* and those in *social rented* housing); and finally, the locale in which the respondent lived (*urban*, *larger towns* and *rural* areas). Also asked were questions on residential attributes: how long the respondent lived in their area of residence (less than 10 years; 10–19, 20–39, and 40 or more years); and whether they had ever lived outside NI for more than six months (*never outside NI*; lived in *Great Britain or the Republic of Ireland*, or *lived elsewhere*). Several questions were included on religion and religious observance: religion stated (*yes*, *no*); religious denomination (*Catholic*, *Protestant*, *Other*); and church attendance (*at least weekly*, *monthly*, *yearly*, or *never attend*).

### Measures

Ten questions related to personal experience and knowledge of dementia, personal attitudes to dementia and perceived public perceptions. These are detailed below [28,2]. Two questions examined the form and intensity of respondent contact with people with dementia: (a) on level of contact—none, *job-related*, *within family* or as part of *some wider circle of acquaintance*; and (b) for those recording such contact, on the level of caring experience or assistance they might have given. For analysis purposes these were recoded as: contact (*none*, contact *within family/friends*, or *amongst acquaintances*); and experience of caring (*none*, direct *experience of caring*, or *offering other assistance*).

Knowledge of dementia was examined across two dimensions: as (a) more general or tacit background knowledge (is it a *disease of the brain*, a *mental illness*, part of *normal ageing*, or *another term for Alzheimer’s*), and (b) more specifically, associated risk factors (is there a *genetic component*, is it related to *heavy smoking*, *poor diet*, *high blood pressure* or *excessive alcohol consumption*). The responses to each set of questions were aggregated into three categories (*agreement* with the statement, *disagreement*, or *no opinion* offered) and each separately further summarised: for (a) as the *number of correct answers* to the questions; and for (b) as four or more incorrect, three incorrect, a mixed response (for the five answers in the ratio 2-2-1), three correct and four or more correct.

Negative and Positive attributions of dementia: respondents were provided with thirteen words relating to dementia (*unpredictability*, *fun*, *gentility*, *fear*, *confusion*, *lost*, *kind*, *dangerous*, *trapped*, *happy*, *angry*, *sad*, *pathetic* or *other*—each allowing only *yes/no (0/1)* responses) and asked to select those most representative of the *way someone who has had dementia for a long time appears* to them. Using this we derived a typology of attitudes to dementia based on the patterns found in the word associations, and represented in terms of levels of p*ositivity or negativity* towards the state of dementia.

Perceptions of Dementia recorded the level of respondent agreement with statements on their perceptions of dementia, in terms of both personal autonomy and treatment of those with dementia, and depletion of their character or personhood.

Perceptions of control or personal autonomy: a measure of how much personal autonomy respondents thought it appropriate to afford people with either early-stage or late-stage dementia. This was based on four questions—should *they continue to live unaided*, control their *own medication*, continue to *drive a car* or *wear an electronic tag*: with responses summarised as *agree*, *disagree* and *no opinion* offered. All except the information on electronic tags were combined and a composite variable derived quantifying the extent to which respondents thought it was appropriate to curtail or control the personal autonomy of those with dementia: with values (a) *low* levels of control appropriate; (b) *no opinion* offered; and (c) *high* levels of control necessary.

Caregiving in dementia: examined respondent opinion on the effects of caring for someone with dementia–whether it can be *lonely*, *rewarding*; or can *affect caregiver’s health*. This was summarised as a single variable: respondents *agree*, *disagree* or record *no opinion* on what they perceive to be the effect of caring for those with dementia.

### Statistical analysis

We described the main socio-demographic features of the respondent cohort, their personal experiences, perceptions and background knowledge of dementia and examined these in relation to the main outcomes (outlined above) using multinomial logistic regression. Using the 13-word item question, we undertook latent class analysis (LCA), deriving a summary three term classification of respondent perceptions of dementia, as captured by the word associations–here the LCA technique groups the participants on the basis of similar response patterns in their selection of the word associations, and generates new indicator variables based on these. The selection of an optimal latent class solution (or *Best Fit*) is evaluated using a standard set of fit statistics: the standard Akaike Information Criterion (AIC) and the Bayesian Information Criteria and its sample-size adjusted version (BIC & SaBIC), as well as the Mendell-Rubin Likelihood Ratio Test; with the model selection also assessed in line with both substantive rationale and background theory). Finally, logistic regression was used to examine factors associated with attitudes to dementia. The main analysis was undertaken using Stata 13 [29], with Mplus [30] used for the LCA.

## Results

Of 2,161 potentially eligible respondents 481 could not be located and 469 declined involvement, leaving 1,211 respondents, a response rate of 56%, similar to earlier studies [31]. Table 1 shows selected socio-demographic and socio-economic characteristics for the respondent group, with column 5 comprising a set of tabulations derived from the Northern Ireland Longitudinal Study [32] (and see note at the foot of Table 1). While these while are presented as indicative only the majority of the indicators compare well to that of the study population: two exceptions being the older age structure of the respondent population when compared to the Northern Ireland population; and the distribution of housing tenure with fewer of the respondent population in owner occupation and correspondingly more in social rented accommodation.

**Table 1:** Main socio-demographic and socio-economic characteristics, by level of contact with people with dementia.

		None recorded % (n)	Contact within family/friends % (n)	Wider circle of acquaintance % (n)	All % (n)	Data^$^ representative of NI population %(n)
Gender	Male Female	50.1 (237) 49.9 (236)	38.3 (150) 61.7 (242)	42.2 (146) 57.8 (200)	44.0 (533) 56.0 (678)	47.8 52.2
Age	18–34 35–54 55–74 75 plus	34.0 (161) 33.8 (160) 21.6 (102) 10.6 (50)	24.6 (96) 34.0 (133) 32.0 (125) 9.5 (37)	13.3 (46) 29.0 (100) 39.7 (137) 18.0 (62)	25.1 (303) 32.5 (393) 30.1 (364) 12.3 (149)	30.6 36.0 25.7 7.8
Tenure	Owner occupier Private renting Social renting	53.0 (249) 24.7 (116) 22.3 (105)	73.5 (288) 10.7 (42) 15.8 (62)	65.5 (226) 14.2 (49) 20.3 (70)	63.2 (763) 17.2 (207) 19.6 (237)	74.0 14.2 11.8
Locale	Urban Rural	59.4 (281) 40.6 (192)	77.8 (305) 22.2 (87)	59.5 (206) 40.5 (140)	65.4 (792) 34.6 (419)	63.9 36.1
Religion	Catholic Protestant Other	44.4 (208) 39.0 (183) 16.6 (78)	42.8 (166) 51.8 (201) 5.4 (25)	45.6 (155) 49.1 (167) 5.3 (18)	44.0 (529) 45.9 (551) 10.1 (121)	41.9 47.4 10.7
Social class	Professional Intermediate (Semi) routine Other	15.9 (75) 26.4 (125) 39.3 (186) 18.4 (87)	31.9 (125) 26.3 (103) 31.4 (123) 10.5 (41)	23.1 (80) 28.6 (99) 35.8 (124) 12.4 (43)	23.1 (280) 27.0 (327) 35.8 (433) 14.1 (171)	26.7 23.1 37.8 12.5
Education	Higher Intermediate No qualifications	21.6 (102) 54.8 (259) 23.7 (112)	34.2 (134) 48.5 (190) 17.4 (68)	25.1 (87) 46.5 (161) 28.3 (98)	26.7 (323) 50.3 (610) 23.0 (278)	29.1 41.9 29.0
Personal experience of caring	None Caring: yes Other help	100.0 (473) - -	18.1 (71) 39.0 (153) 42.9 (168)	32.7 (113) 14.7 (51) 52.6 (182)	54.3 (657) 16.9 (204) 28.9 (350)	
General knowledge of dementia	Low (0/1 correct) High (2 or more)	29.8 (141) 70.2 (332)	41.6 (163) 58.4 (229)	38.4 (133) 61.6 (213)	36.9 (431) 63.9 (774)	
Knowledge of risk factors	Mostly incorrect Mostly correct	78.4 (371) 21.6 (102)	79.1 (310) 20.9 (82)	77.2 (268) 22.8 (79)	78.3 (948) 21.7 (263)	

^$^: column 6 was derived from tables produced using the Northern Ireland Mortality (NIMS)–a database comprising the whole of the enumerated 2011 Census population of Northern Ireland, and part of the Northern Ireland Longitudinal Study (NILS). More detail can be found at https://www.qub.ac.uk/research-centres/NILSResearchSupportUnit/. Where the cell is empty it was not possible to tabulate equivalent results. The findings are used with permission of the Northern Ireland Statistics and Research Agency (NISRA), and we acknowledge their help in producing these results.

### Contact and care-giving

Females and people aged 35–74 were more likely to have contact with people with dementia irrespective of locale, with owner occupiers more likely to report *within-family* contact (74%) than other tenure groups, and Catholics less likely than Protestants to report *within-family* contact (43% and 52% respectively). Of those reporting *within-family* contact, 39% had a caring role and 43% a lesser helping role. Among people with more distant contact 33% reported no experience of caring and 53% provided help other than caring. Generally, while 64% of respondents showed relatively good lay knowledge of dementia, 78% incorrectly answered the more specific risk-factor questions (Table 1). A substantial proportion of people cited *having no opinion* (ranging from 39% to 56% for each question), and 56% of respondents wrongly answered four (of five) questions asked. Only 11% answered four or more correctly (Table 2).

**Table 2:** Respondent knowledge of risk factors.

Risk factors for dementia..	No opinion offered^&^ % (n)	Agreed % (n)	Disagreed % (n)
high blood pressure familial (or genetic) heavy smoking^£^ poor diet heavy alcohol drinking	55.8 (674) 38.9 (470) 45.8 (554) 41.7 (504) 40.5 (489)	20.3 (245) 33.8 (408) 22.7 (274) 27.7 (335) 32.8 (397)	23.9 (289) 27.4 (331) 31.5 (381) 30.6 (370) 26.7 (323)
Summary—number of questions answered correctly			
4 or more responses correct 3 responses correct mixed answers^$^ 3 responses incorrect* 4 or more incorrect*	11.2 (136) 10.5 (127) 13.4 (162) 8.5 (103) 56.4 (683)		

^£^: The *smoking* question is framed as not being a risk factor–here *agreed* and *disagreed* categories are reversed to allow the correct answer to be stated

^$^: Mixed answers—relationships in the ratio 2-2-1 over the five answers

*: Incorrect responses include those who *offer no opinion*

^&^: Categories associated with the variables were: definitely, probably, no opinion, probably not, definitely not, refused to answer & don’t know. These were summarised as: *no opinion offered*—no opinion, refused to answer & don’t know; *agreed*—definitely & probably); and *disagreed*—definitely not & probably not.

Table 3 records perceptions of the experience of caring for people with dementia and its potential effects on the caregiver. Caregiving was regarded as predominantly burdensome (67%): it can be *lonely* (80%), *unrewarding* (52%) and potentially a *health burden* (71%). Table 4 examines respondent perceptions of how dementia affects those diagnosed—with the majority either expressing no opinion or espousing statements highlighting aspects of loss-of-self (44.6% and 49.4% respectively).

**Table 3:** Perceptions of health burden on those caring for people with dementia.

*Caring for someone with dementia can* ..	No opinion offered& % (n)	Agree with statement % (n)	Disagree. % (n)
.. be very lonely .. be very rewarding* .. lead to own health suffering summary: caring for someone with dementia places a burden on those providing it	15.8 (191) 26.7 (323) 20.6 (249) 24.2 (293)	79.6 (962) ***21*.*7 (262****)* 70.8 (856) 67.4 (816)	4.6 (56) ***51*.*6 (624)*** 8.6 (104) 8.3 (100)

*: adjusted to reflect the orientation of the original question.. i.e. in the summary figure the underlined figures are exchanged

^&^: please refer to the notes at the foot of Table 2

**Table 4:** Respondent perceptions of the personality changes seen to be part of a patient’s dementia pathway.

*For people with dementia*.. *there comes a time when* ..	Agree with statement % (n)	Disagree with statement % (n)	Unsure/don’t know.. % (n)
.. all you can do is keep them *clean*, *healthy & safe* .. *others make decisions* for those with dementia too much .. ..the *person disappears* .. the person *ceases to be treated as human* .. life is *not worth living* .. they are *treated like children*	76.8 (930) 49.4 (598) 72.8 (881) 50.0 (605) 34.6 (419) 64.3 (779)	11.9 (144) 18.3 (222) 9.5 (115) 21.7 (263) 39.5 (478) 14.4 (174)	11.3 (137) 32.3 (391) 17.8 (215) 28.3 (343) 25.9 (313) 21.3 (258)
Summary: respondent mainly in agreement with the statements respondent mainly disagreed with the statements respondent expressed mixed opinions–non-decisive respondent mainly expressed no opinion	% (N) 49.4 (598) 6.0 (73) 34.3 (415) 10.3 (125)		

### Attributes of dementia

**Fig 1:**
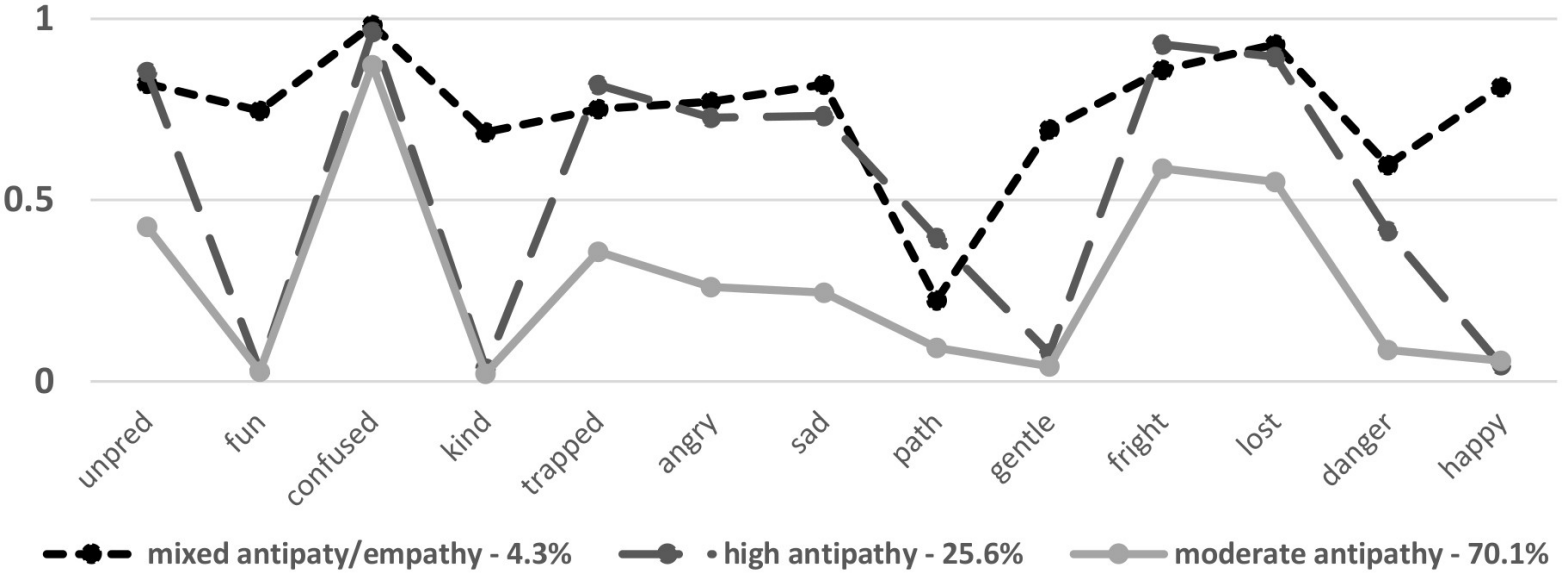
Latent class analysis of attributes of dementia.

Relationships between thirteen putative dementia attributes elicited more complicated responses: eighty respondents didn’t answer and thirteen included all attributes—these were excluded, leaving 1118 for analysis. The most commonly chosen attribute was *confused*, cited by over 90% of respondents and also most consistently selected in conjunction with other items. Generally, more positive or empathetic attributes (*fun*, *gentility*, *kind* and *happy*) correlated highly with each other but lowly with attributes classifiable as more negative. The term *pathetic* was cited least in conjunction with the other attributes (ranging from 17% to 30% over each) and the term *dangerous* was cited most often in conjunction with attributes eliciting empathy (range = 36%-46%). We used LCA to establish relationships between these attributes—Fig 1 shows the emergent three-class solution, indicating the feelings involved in thinking about the lived-situation of people with dementia: a small group reporting mixed positive/negative feelings (4% of respondents (n = 50)); a group recording high levels of negativity (26%); and a larger group recording more moderate levels of negativity (70%). The groups exhibiting moderate attitudes were combined producing a binary outcome: those with moderate attitudes, and those describing high levels of negativity (0/1). Table 5 shows the relationship between these expressed negative attitudes, contact with people with dementia and knowledge of dementia. In the fully adjusted model: females more likely than males to express negative attitudes (Odds Ratio (OR) = 1.71: 95%CI = 1.28, 2.29); similarly, those in urban areas compared to their rural counterparts (OR = 1.42: 1.05, 1.94); while no association was recorded with age (however, those in the eldest age group recording lower likelihoods when compared with those younger). Respondents with higher levels of general knowledge of dementia and those less knowledgeable about risk factors were more likely to express higher levels of negativity (OR = 2.51: 1.64, 3.83 and OR = 1.71: 1.19, 2.46 respectively) than their comparator groups, as were those who had personal contact, compared with no contact (OR = 1.59: 1.01, 2.50 for when within a *wider circle of acquaintanceship)*.

**Table 5:** Factors associated with higher levels of negative feeling (or antipathy) to dementia. Data represents Odds Ratios (and 95% Confidence Intervals).

		outcome = high levels of negative feeling about dementia (versus more moderate attitudes)	
		univariate analysis: OR (95%CI)	fully adjusted: OR (95% CI)
gender	male female	1.00 1.73 (1.31, 2.29)***	1.00 1.71 (1.28, 2.29)***
age group	18–34 35–54 55–74 75 plus	1.00 1.08 (0.75, 1.55) 1.09 (0.75, 1.58) 0.81 (0.49, 1.33)	1.00 1.01 (0.69, 1.47) 1.00 (0.67, 1.47) 0.72 (0.43, 1.22)
locale	rural urban	1.00 1.10 (0.83, 1.46)	1.00 1.42 (1.05, 1.94)*
contact with people with dementia	none recorded yes: within family yes: wider circle of acquaintance	1.00 1.70 (1.22, 2.38)** 1.87 (1.34, 2.64)***	1.00 1.35 (0.83, 2.17) 1.59 (1.01, 2.50)*
personal experience of caring for people with dementia:	none recorded yes: caring experience yes: other experience	1.00 1.56 (1.09, 2.25)* 1.56 (1.15, 2.12)**	1.00 1.24 (0.77, 2.01) 1.19 (0.79, 1.81)
more general knowledge of dementia:	mainly incorrect answers mainly correct answers	1.00 2.54 (1.69, 3.84)***	1.00 2.51 (1.64, 3.83)***
knowledge of risk factors:	mostly correct mostly incorrect	1.00 1.58 (1.12, 2.23)**	1.00 1.71 (1.19, 2.46)**

***: p = 0.000;

**: p<0.001;

*: p<0.05

### Autonomy and control

Eighty per cent of respondents thought it appropriate that high levels of control be exerted on those with late-stage dementia, with 43% regarding it as appropriate even at early-stage (Table 6). Those offering *no opinion* to these questions also followed a pattern–higher proportions offered *no opinion* to early-stage than late-stage scenarios (39% and 18% respectively). Most respondents were disinclined to allow people with dementia to drive (59% and 84% for early-stage and late-stage respectively), and most considered electronic tags necessary in both early- and late-stage scenarios (53% and 57% respectively).

**Table 6:** Attitudes to dementia: Degree of independence which should be afforded to those with (a) late stage and (b) early stage dementia.

.. *person with dementia should be allowed to*..	No opinion offered^&^ % (n)	Agree with low control % (n)	Agree with high control % (n)
***Late stage dementia*:** continue living alone control own medication drive car Summary: independent living wear electronic tags	20.9 (253) 17.5 (211) 14.8 (179) 18.1 (219) 23.3 (282)	3.1 (37) 2.7 (32) 1.7 (20) 2.2 (26) 19.4 (235)	76.0 (919) 79.9 (966) 83.5 (1010) 79.7 (964) 57.2 (692)
***Early stage dementia*:** continue living alone control own medication drive car Summary: independent living wear electronic tags	42.9 (519) 37.1 (449) 31.9 (386) 39.4 (476) 28.7 (347)	26.6 (322) 19.8 (239) 9.4 (114) 17.9 (216) 18.7 (226)	30.4 (368) 43.1 (521) 58.6 (709) 42.8 (517) 52.6 (636)

^&^: as with Table 2 above

Rural residence, lower education and age were associated with toleration of *tagging*, even in early stage dementia (Table 7). Conversely, those with personal contact and experience of caring, and knowledge of dementia were less likely to advocate tagging. Columns 5&6 highlight factors associated with acceptance of the need for greater control of people with early-stage dementia. Early middle-aged respondents (35–54) were less likely to advocate control (OR = 0.55: 95%CI = 0.35, 0.87), and those less well-educated more likely, compared to people with degree-level qualifications (OR = 1.56: 1.07, 2.28 and OR = 2.98: 1.76, 5.03 for intermediate-level and no qualifications respectively); and those with more limited knowledge of risk factors (OR = 2.75: 1.88, 4.02). Columns 3&4 highlight factors associated with advocacy of *electronic tagging* for persons with early-stage dementia: with those from urban areas less in favour when compared to their rural counterparts (OR = 0.55: 95%CI = 0.40, 0.78); and those less well-educated compared to those educated to degree-level (OR = 1.56: 1.10, 2.22 and OR = 2.18: 1.34, 3.54 for those with intermediate levels and no qualifications respectively). Finally, the patterns outlined above also broadly apply to the factors associated with those recording no opinion or mixed responses.

**Table 7:** Factors associated with levels of control/autonomy thought acceptable for those with early-stage dementia^$^. Data represents Odds Ratios (and 95% Confidence Intervals) from fully adjusted models.

		use of electronic tags (reference = more relaxed attitudes to tags)		more general markers of control/autonomy (ref = moderate levels of control/autonomy)	
		unsure/no opinion OR (95%CI)	in favour of using electronic tags: OR (95% CI)	mixed answers or no opinion OR (95%CI)	more prescriptive on need for control: OR (95% CI)
gender	male female	1.00 0.90 (0.63, 1.29)	1.00 1.07 (0.78, 1.47)	1.00 0.89 (0.63, 1.24)	1.00 1.14 (0.81, 1.60)
age group	18–34 35–54 55–74 75 plus	1.00 0.66 (0.42, 1.04) 0.44 (0.27, 0.73)** 1.03 (0.52, 2.05)	1.00 1.16 (0.76, 1.76) 1.05 (0.68, 1.63) 1.50 (0.79, 2.84)	1.00 0.51 (0.33, 0.80)** 0.53 (0.32, 0.86)* 0.63 (0.33, 1.22)	1.00 0.55 (0.35, 0.87)* 0.87 (0.53, 1.43) 0.81 (0.42, 1.56)
locale	rural urban	1.00 1.06 (0.73, 1.54)	1.00 0.55 (0.40, 0.78)**	1.00 1.08 (0.75, 1.56)	1.00 0.71 (0.49, 1.02)
highest educational attainment	degree intermediate no qualifications	1.00 1.50 (1.00, 2.24) 2.16 (1.24, 3.75)**	1.00 1.56 (1.10, 2.22)* 2.18 (1.34, 3.54)**	1.00 1.43 (0.98, 2.08) 1.78 (1.03, 3.06)*	1.00 1.56 (1.07, 2.28)* 2.98 (1.76, 5.03)***
contact with people with dementia	none recorded yes: within family yes: wider circle	1.00 0.37 (0.20, 0.69)** 0.55 (0.31, 0.97)*	1.00 1.02 (0.60, 1.73) 1.06 (0.64, 1.76)	1.00 0.57 (0.31, 1.05) 0.75 (0.43, 1.32)	1.00 1.22 (0.68, 2.21) 1.36 (0.78, 2.37)
personal experience of caring for people with dementia:	none recorded yes: caring experience yes: other experience	1.00 1.64 (0.84, 3.23) 0.88 (0.50, 1.56)	1.00 1.28 (0.73, 2.26) 0.99 (0.62, 1.59)	1.00 1.23 (0.65, 2.34) 0.89 (0.51, 1.55)	1.00 1.06 (0.57, 1.95) 0.81 (0.48, 1.38)
more general knowledge of dementia:	mainly incorrect answers mainly correct answers	1.00 0.45 (0.29, 0.70)**	1.00 0.72 (0.46, 1.10)	1.00 0.87 (0.58, 1.31)	1.00 1.24 (0.81, 1.89)
knowledge of risk factors:	answers mainly correct .. mainly incorrect	1.00 1.52 (0.98, 2.36)	1.00 0.92 (0.63, 1.34)	1.00 2.64 (1.81, 3.85)***	1.00 2.75 (1.88, 4.02)***

***: p = 0.000;

**: p<0.001;

*: p<0.05

^$^: level of control derived by summarising the elements of question five—should people with early-stage dementia (1) continue to live alone; (2) continue to drive a car; and (3) administer their own medication

## Discussion

Despite much public health effort—and media interest—in communicating to the public that dementia progression may be amenable to *modifiable risk factors* [33,34,1,35] these findings confirm continuing entrenched levels of negativity amongst the general public [2–4]: with higher levels associated with better general or tacit knowledge; and more specific knowledge of risk factors mitigating somewhat against it. We note that, in this study, antipathy to dementia, which can be thought of as a precursor of stigma, is inversely associated with knowledge and contact. Moreover, respondents indicated they thought high levels of control were appropriate for people with diagnosed, even with early-stage dementia. These findings highlight the complexity of social and personal responses to dementia. Thus, knowledge about dementia and personal contact with someone who has dementia do not always engender sympathetic responses. Perhaps more in keeping with evidence from elsewhere, older age, lower education and limited knowledge are associated with perceptions of the need to exert high levels of control over people with dementia, even at early stages. Nevertheless, the findings highlighting negative characterisation of dementia and the perceived need for surveillance and control raise interrelated questions about how to address public anxiety. Are such fears related to a concern for individuals with dementia (and their social context) or do they drift into a wider social response reflecting stigmatising attitudes? Stigma is, necessarily, understood and constructed through social relationships and the rules that govern *normality* or what is expected [36]. However, dementia-related stigma remains relatively untheorized [37].

While dementia services are exhorted to overcome what Kitwood [38] termed a malignant social psychology, the social exclusion of people living with dementia is still prevalent and this is likely to continue while families and communities lack resources to produce change. To what extent a sense of shame, embarrassment about dementia symptoms, fear of accidents, or difficulties in negotiating social barriers are risk factors for exclusion is unclear. Thus, while it is important to distinguish between actual and the psychological barriers to social engagement there is a likely interaction between the inherent challenges of dementia, the anticipated problems by the patient or family caregiver, and the environmental capacity to deal with them, that reinforces a ‘housebound’ exclusion. While this persists, contact with people with dementia will remain limited and public dementia anxiety high.

### Strengths and limitations

This is a cross-sectional study and therefore no conclusions can be drawn about causation. While the measures and questions that *tap* into attitudes to dementia are not validated, it is important to note that there are no well-established and validated measures of dementia-related stigma. Moreover, the questions asked do provide sufficient breadth for the attitudes examined to be sufficiently nuanced for analysis.

### Conclusion

In this examination of attitudes to dementia we found that respondents emphasised its challenging nature, reporting high levels of negativity across a range of issues. The study also highlighted a lack of knowledge of risk factors about dementia. Given that the underlying dementia knowledge-base is rapidly developing, with both a research focus on the modifiability of risk factors, and a shift towards a more dynamic open debate about all aspects of the disease, it seems appropriate that future attitudinal research should reflect this and focus more attention on how this *cultural* shift can be realized and what psychological and institutional barriers still remain within this current more fluid situation. More research is needed to establish effective strategies to increase knowledge about dementia in the lay population. Strategies to facilitate stronger social inclusion for people with dementia and their carers should be a priority for research and policy.
